# Training deaf college students to improve their theory of mind: based on a two-component model

**DOI:** 10.3389/fpsyg.2024.1361878

**Published:** 2024-04-17

**Authors:** Yang Wu, Xiping Liu, Shengnan Zhang

**Affiliations:** ^1^School of Marxism, Tianjin University of Technology, Tianjin, China; ^2^Faculty of Psychology, Tianjin Normal University, Tianjin, China; ^3^Faculty of Education, Henan Normal University, Xinxiang, China

**Keywords:** training, deaf Chinese college students, theory of mind, cognitive psychology, development psychology

## Abstract

This paper explored the training methods to improve the level of deaf college students’ ToM. Eighty deaf college students were selected as participants and randomly divided into experimental group and control group. The ToM training group received ToM training; The non-ToM training group received physical-conversation training. Cognitive ToM task and affective ToM task were used to investigate the training effect. After training, the level of ToM of deaf college students who received ToM training was significantly improved. The results show that ToM training can effectively promote the level of deaf college students’ ToM.

## Introduction

Theory of Mind (ToM) is the ability to make inferences about the psychological states of others and predict or explain their behavior with reference to their mental states, feelings, beliefs, and desires (Premack and Woodruff, 1978; Wellman and Liu, 2004). ToM is a key factor in the development of interpersonal skills and the formation and maintenance of social relationships (Devine and Hughes, 2016; Lecciso et al., 2016; Petrocchi et al., 2021).

Studies of deaf preschool children and deaf school-aged children have found that ToM delay can persist, at least in some respects, until the age of 12 or 13 years (O’Reilly et al., 2014). In a study of social perception, Lecciso et al. (2013) measured this component of ToM using the Eye Test task (Baron-Cohen et al., 2001) and found that deaf children, aged 5–14 years, performed worse than children with normal hearing. Although ToM of deaf individuals with deaf parents may equal that of hearing individuals later in development, evidence of delayed development after adulthood remains for deaf individuals without deaf parents (O’Reilly et al., 2014). A study evaluated deaf college students’ understanding of sarcasm, second-order false beliefs, and double deception. The results showed that these individuals scored significantly lower on all three ToM tests than their hearing peers. The same results were found in Edwards et al. (2021), where deaf college students performed worse than their hearing peers on sarcasm, metaphor comprehension tasks, and reasoning tasks.

The reason for this may be that deaf people with hearing parents experience less conversation at home than deaf children with deaf parents (Peterson and Slaughter, 2006). Parents’ conversations with deaf children lack high-level facilitative discourse, such as mental state conversation, and are more indicative than those with hearing children (Ambrose et al., 2015). This situation results in deaf children often having no one at home with whom they can freely talk about observed thoughts, feelings, and other mental states. This development delay indirectly affects deaf college students’ social ability and the formation of social relationships. Studies have shown that a decline in the ability to understand mental states may be positively correlated with a decline in social activities (Bailey et al., 2008), and negatively correlated with the scale of intimate social networks (Radecki et al., 2019). Deaf college students who are about to enter the society are at a special stage. They will soon independently bear social and personal responsibility; therefore, they often demonstrate a need improve their level of ToM and social adaptability during this critical transition. Therefore, it is very important to find ways to quickly improve their theory of mind. In view of the previous studies, ToM can be divided into cognitive ToM and affective ToM, so this study hopes to find a training method that can improve the cognitive ToM and affective ToM of deaf college students at the same time, so as to help deaf college students improve their social adaptability.

### Factors influencing training

Thus far, studies promoting ToM levels have typically been conducted on preschoolers, school-age children, individuals with autism, and older individuals (Wellman et al., 2001; Caputi et al., 2021). Training studies in older adults have shown that ToM training based on mental state conversations is effective in improving social cognitive skills in nursing home residents. Older people are able to transform the skills acquired during training into new materials, thereby improving their quality of life (Cavallini et al., 2021). Such training also shows the importance of mental state conversations in promoting ToM levels.

In addition to the mental state conversations, there are other details that can be focused upon when training. The first is whether reading materials with a large number of mental state words are included in the training process. In adults, Kidd and Castano (2013) demonstrated the effect of reading literary fiction on ToM skill development. People who read literary fiction performed better on a ToM task than those who read non-fiction. This association may be a function the large number of words related to ToM typically encountered in novels. In addition, the researchers found that mothers’ use of mental state words while reading picture books was strongly correlated with their children’s performance on false belief tests, and this association lasted for at least 1 year (Adrian et al., 2007). In the process of reading, the reader is required to make inferences about the characters and the plot of the story, and to understand the vocabulary of mental states used in the story, all of which support ToM development (De Mulder et al., 2017).

The second training detail is whether there is a large amount of interpretation in the training process. Research on ToM training shows that informing a child whether their answer is correct and explaining why it is necessary can make a difference in ToM ability (Clements et al., 2000; Melot and Angeard, 2003). Giving feedback and explanation in conversations can make a connection between a character’s inner state and outward behavior (Slaughter and Peterson, 2012). For example, Schult and Wellman (1997) found that preschool children explain more than 85% of voluntary behavior and mistakes made by others through their mental states, and that explanation can help them build their personal worldview.

The final training detail is a focus on facial cues. While individuals without hearing loss rely heavily on facial changes as supplementary cues to comprehend conversational content, for deaf individuals, this is a crucial factor in communication. Furthermore, native sign language places greater emphasis on the eye area (Emmorey et al., 2009), while late sign language learners tend to focus more on the mouth area (Muir and Richardson, 2002). Therefore, facilitating easier observation of facial cues during training is highly important for deaf individuals.

### Current study design

ToM training utilized in this study was based on the comprehensive ToM treatment approach developed by Cavallini for older adults. First, during the training process, participants engaged in group discussions. Participants were arranged to sit in a circle, enabling them to visually perceive each other’s facial expressions and sign language cues synchronously. It is worth noting that most sign languages have distinct mouth shapes associated with gesture movement and timing (Boyes Braem and Sutton-Spence, 2001; Sutton-Spence, 2007). Second, an extensive collection of reading materials containing mental state vocabulary was prepared for the training sessions. These materials encompassed ToM state verbs which are particularly focused on abstract internal states and cognitive processes. A study by de Villiers and de Villiers (2014) found that employing mental state verbs directly distinguishes mental content from reality and plays a central role in understanding false beliefs. Finally, ample explanations were incorporated into the training. Interpretation serves as a fundamental aspect of cognition and occurs naturally within everyday social interactions (Keil, 2006). In discussions revolving around mental states, feedback and interpretation appear to be crucial elements (Clements et al., 2000). Throughout this study’s training process, participants were required to explain trainers’ questions, as well as address any doubts, while providing their own answers. Through this interpretive process, we hypothesized that participants would enhance their self-understanding and comprehension of the world, including ToM concepts.

In addition, previous research designs have used different ToM stories to examine the overall effect of training. These testing methods are unable to examine multidimensional changes in ToM and compare differences between dimensions. The current study attempts to investigate training effects on the cognitive and affective dimensions of ToM based on the two-component model. Some researchers believe that ToM is composed of two parts: cognitive processing and emotional processing (Shamay-Tsoory et al., 2006). Brothers and Ring (1992) believe that cognitive ToM, which refers to the inference of knowledge and belief, develops around 4–5 years old. Affective ToM, which refers to the inference of emotion, is thought to develop around 2–4 years old (MacDonald et al., 1996). Many studies have shown that affective ToM and cognitive ToM can be separated in behavior (Hynes et al., 2006; Völlm et al., 2006; Shamay-Tsoory and Aharon-Peretz, 2007). Extensive studies using brain imaging techniques have simultaneously demonstrated the existence of a partial separation mechanism at the neural level between cognitive and affective ToM (Dvash and Shamay-Tsoory’s, 2014). This neuroanatomical separation between affective and cognitive regions has also been shown in deaf individuals (Hao et al., 2010; Lecciso et al., 2016). This study examines the theory of mind level of deaf college students from two component and further verifies the existence of separate component from the results.

Finally, previous training studies have often used test materials containing text to examine training effects, which easily adds additional processing burden to the deaf group in completing the task, possibly reducing the level of ToM assessment. In this study, two picture only tasks were selected, which could be performed without relying on text processing, and the effect of training could be objectively judged.

Therefore, this study takes mental state as the focus of ToM training, and combines the above factors affecting ToM changes to develop suitable training content for deaf college students, in an attempt to improve the ToM level of deaf college students through four training courses. Cognitive and affective ToM levels of deaf college students before and after training were investigated through two experimental procedures, each focusing on cognitive or affective ToM. Two months after the training, cognitive ToM and affective ToM were tested again to investigate the long-term effects of the training.

## Methods

### Participants

A total of 80 deaf college students (*M* = 20.15 years old, SD = 1.34; 50%, female) were recruited through information provided on the website of a special education university in Northern China. Participants were required to be 18 years of age or older and have hearing loss. Participants were also required to pass a university entrance exam that included reading and writing content, and demonstrate a proficient level of written and spoken Chinese or Chinese Sign language. None of the students were clinically diagnosed with any mental disorder. An intelligence test was completed by participants prior to group assignment, and participants did not receive any tangible reward (such as money or gifts). Participants were randomly assigned to two groups: the ToM training group (18–22 years old) and the no-ToM training group (18–22 years old). There was no significant difference in age [*F*_(1, 78)_ = 1.60, *p* > 0.05], grade [*F*_(1, 78)_ = 0.35, *p* > 0.05], sex [*F*_(1, 78)_ = 0.13, *p* > 0.05], or intelligence [*F*_(1, 78)_ = 2.50, *p* > 0.05] between groups. The descriptive statistics of age, grade, intelligence, male to female ratio, and hearing level are shown in Table 1. All participants completed an informed consent form before starting the study.

**Table 1:** Participant characteristics.

	**Participant characteristic**	**ToM training (*n* = 40)**		**No-ToM training (*n* = 40)**	
		***M***	***SD***	***M***	***SD***
	Age	20.25	1.14	20.05	1.53
	Raven	40.20	3.21	39.75	3.72
Female, *n*		21		19	
Grade, *n*	Freshman	14		13	
	Sophomore	13		14	
	Junior	13		13	
The hearing status, n	Severe hearing loss	10		13	
	Profound hearing loss	26		22	
	Complete or total hearing loss/deafness	4		5	

Scores in parenthesis refer to standard deviation; Parental education level: 1 = no/primary education; 2 = lower general secondary education; 3 = higher general secondary education; 4 = college/university; Grades of hearing loss is based on the grading system published by WHO in 2021.

### Measures and procedure

#### Research design

This study used a 2 (group: ToM training vs. no ToM training) × 3 (timepoint: pre-test, post-test 1, post-test 2) two-factor-mixed experimental design. Group was the between-subject variable and timepoint was the within-subject variable.

#### Intelligence test

Raven’s Reasoning Test is a kind of nonverbal intelligence test, which was compiled by Raven in 1938, and revised by Zhang and Wang (1985), which established Chinese urban norms. The test consists of five series, each consisting of 12 questions, all of which are graphic reasoning questions. The standard version of the test contains 60 items. Each item consists of a large graph and several small graphs. The lower right corner of the large graph is missing. The task of the test subjects is to find out which part is missing in the lower right corner of the large graph. The test is scored on a 2-point scale, meaning correct answers are scored as 1 point and incorrect answers are scored as 0 points. The partial reliability of the test was 0.95, 0.82 for retest at half month intervals, and 0.79 for retest at 1 month intervals, indicating good reliability. The prediction validity is good.

#### Cognitive ToM

**Figure 1:**
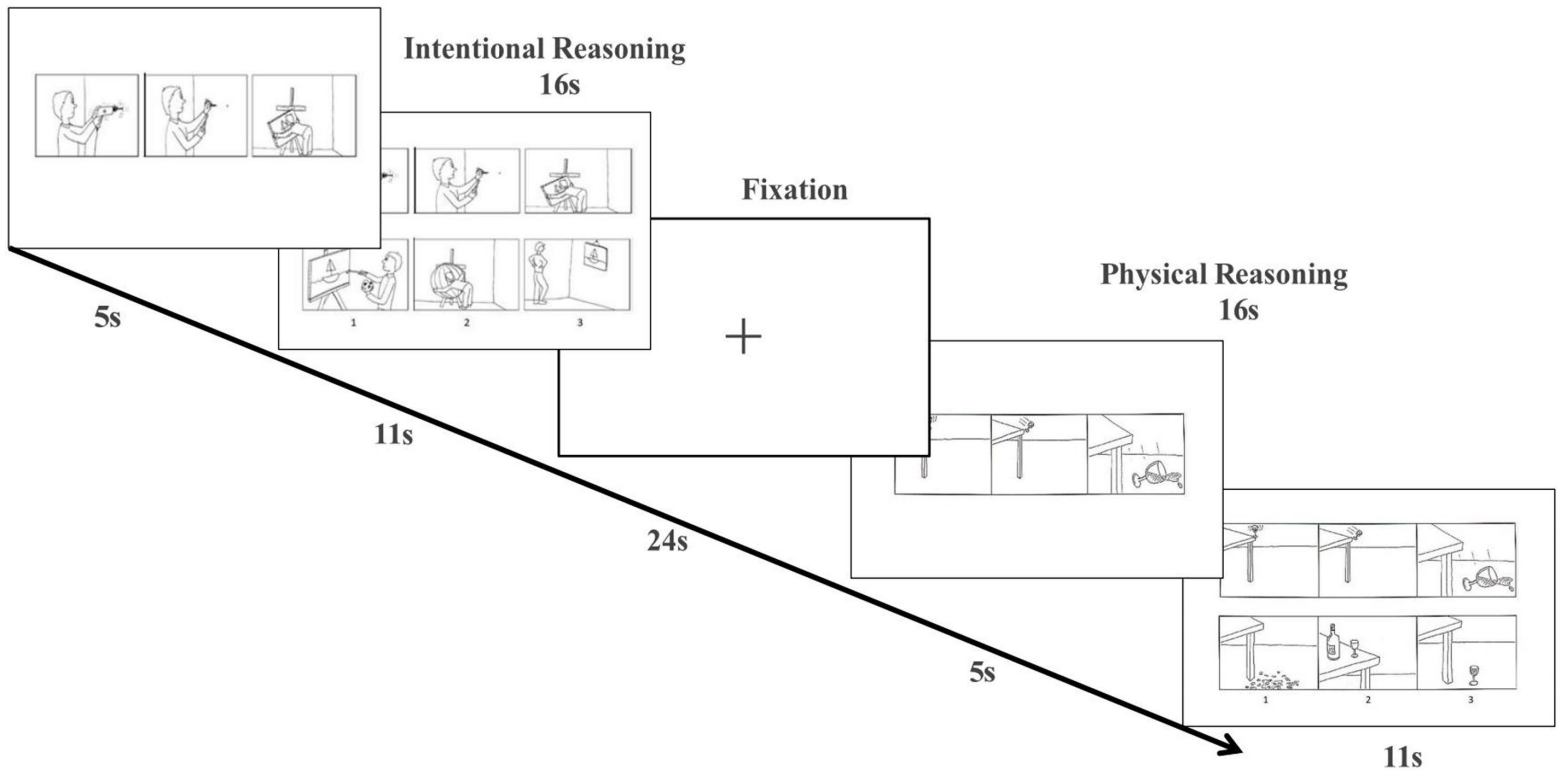
Cognitive ToM task. Task design and example stimuli. The correct answers for the example stimuli pictured here are “3” (intentional condition) and “1” (physical condition). s, seconds.

A total of 36 items were presented to participants (18 were intentional reasoning tasks and 18 were physical reasoning tasks) (Murdaugh et al., 2014; Thye et al., 2018). In the intentional reasoning task, participants were shown a comic strip with three images of objects or people and were asked to choose a logical ending from three options. In the physical reasoning task, subjects were presented with a cartoon image depicting a physical behavior, while in the intentional reasoning condition, subjects were presented with three images depicting goal-oriented and social interaction behavior, with specific presentation modes (Figure 1).

#### Affective ToM

**Figure 2:**
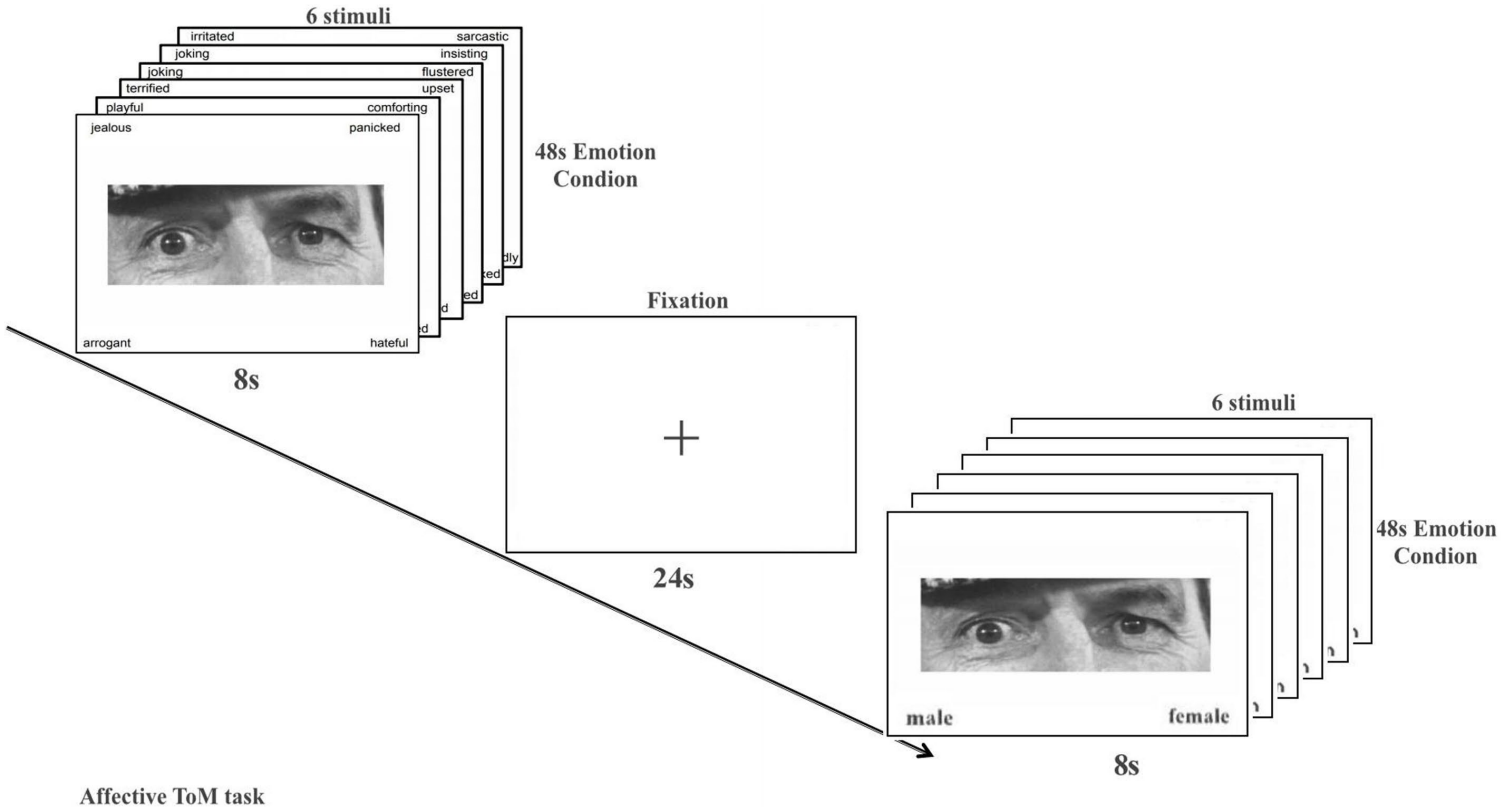
Affective ToM task. Task design and example stimuli. The correct answers for the example stimuli pictured here are “panicked” (emotion condition) and “male” (gender condition). s, seconds.

In a revised version of the eye task, participants were presented with the eye area of a face and instructed to choose the most suitable word from a list of four options that best described the mental state conveyed by the character’s eyes. In the control condition, participants were instructed to identify the gender status of the individuals depicted in the photos, with 72 pictures, including 36 that depicted mental states and 36 depicted gender status (Baron-Cohen et al., 2001; Thye et al., 2018) (Figure 2).

### Procedure

Participants were randomly divided into four groups of 20 and trained simultaneously in four classrooms. Two groups received ToM training with conversations focusing on mental states, while other two groups received no-ToM training, with conversations focusing on physical aspects. The training content was adapted from Cavallini’s ToM training program developed in Cavallini et al. (2015), specifically tailored for deaf college students by incorporating classroom, dormitory, and community activity scenes. Each training group had a sign language interpreter to facilitate comprehension of the material. Considering variations in hearing impairment among participants, visual materials were used for presentation purposes.

All participants underwent testing at three timepoints: pre-test, post-test 1, and post-test 2; these assessments were administered by individuals who did not participate in any training. Pre-tests included the Raven Intelligence Test, as well as cognitive and affective ToM tasks. There were four training sessions. The duration of each weekly session was 90 min, and took place over the course of 1 month. The ToM training group was exposed to four stories, each containing conversations about mental states, either imagined or real. Participants were asked to answer questions during the training, and discussed their answers, focusing on the changes in the mental state of the characters in the story and the changes in the mental state of the participants themselves. In each lesson, trainers guided participants through exercises aimed at familiarizing them with mental state verbs and distinguishing them from other types of verbs through practice activities involving substitution within the stories themselves. Each time the no-ToM training group was exposed to the same story, with the only change being questions and discussions based on physical characteristics of the characters. The structure and length of the physical characteristics story was the same as that of the story presented to the ToM training group. The post-training tests took place immediately after training and 2 months after the training, and included the cognitive and affective ToM tests.

### Analytic plan

We hypothesize that ToM training has positive effects on both intentional reasoning and emotional reasoning in the ToM training group, while non-ToM training has positive effects on both physical reasoning and gender reasoning in the non-ToM training group. SPSS 25.0 (IBM Corp., Armonk, NY) was used for statistical analysis. Descriptive statistical analysis and mixed measure ANOVA were used, respectively, to examine the homogeneity of the two groups on different tasks before and after training and the changes before and after training. If the interaction is significant after repeated measurement analysis, simple effect analysis is used to further analyze the results. If the interaction is not significant, the paired *T*-test is used to further analyze the results.

## Results

Table 2 shows the performance of two groups of deaf college students in completing four tasks before and after training. To investigate the effect of training on the performance of the two groups in the cognitive ToM task, a 2 (group: ToM training group, no-ToM training group) × 3 (timepoint: pre-test, post-test 1, post-test 2) mixed measure ANOVA analysis was performed.

**Table 2:** Mean values and standard deviations for task performance as a function of Group Factor (ToM and No-ToM groups) and Timepoint (pre, post-1, and post-2).

				Pre		Post-1		Post-2	
				*M*	*SD*	*M*	*SD*	*M*	*SD*
ToM training	Cognitive ToM	Intentional reasoning	ACC	0.6028	0.13	0.7050	0.11	0.6989	0.13
			RT	4840.1	942.9	4530.8	1027.1	4258.7	948.2
		Physical reasoning	ACC	0.8247	0.14	0.8412	0.14	0.8299	0.14
			RT	4029.6	849.3	3383.3	710.7	3192.1	565.9
	Affective ToM	Emotion reasoning	ACC	0.4757	0.11	0.5417	0.10	0.5348	0.07
			RT	4173.9	557.2	4221.3	552.6	4213.3	556.9
		Gender reasoning	ACC	0.7372	0.10	0.7400	0.04	0.7619	0.08
			RT	2782.6	661.4	2669.2	448.0	2563.1	539.1
No ToM training	Cognitive ToM	Intentional reasoning	ACC	0.6069	0.15	0.6117	0.19	0.6114	0.11
			RT	4494.4	979.3	4266.4	1158.3	4696.4	827.6
		Physical reasoning	ACC	0.8194	0.14	0.8095	0.14	0.8389	0.14
			RT	4261.4	1129.9	4099.2	1111.5	3554.4	784.0
	Affective ToM	Emotion reasoning	ACC	0.5069	0.09	0.5208	0.12	0.5117	0.08
			RT	3891.6	528.2	4147.5	592.87	4019.1	513.2
		Gender reasoning	ACC	0.7380	0.07	0.7675	0.06	0.7823	0.09
			RT	2726.6	473.7	2690.1	507.9	2501.7	489.0

M, mean; SD, standard deviation; ACC, correct rate; RT, reaction time.

Results showed that the accuracy rate (ACC) interaction was significant in intentional reasoning, *F*_(2, 77)_ = 6.12, *p* < 0.05, η^2^ = 0.137. Simple effect analysis showed that there was no significant difference between ToM training group and no-ToM training group for pre-test conditions, *F*_(1, 78)_ = 0.017, *p* > 0.05, η^2^ = 0.000; at the first post-test, the difference between the ToM training and no-ToM training groups was significant, *F*_(1, 78)_ = 7.54, *p* < 0.05, η^2^ = 0.088; at the second post-test, the difference between the ToM and no-ToM training groups was also significant, *F*_(1, 78)_ = 36.27, *p* < 0.05, η^2^ = 0.317. Moreover, there were significant differences in the three measurement results of the ToM training group, *F*_(2, 77)_ = 11.47, *p* < 0.001, η^2^ = 0.23, but no significant differences in the three measurements for the no-ToM training group, *F*_(2, 77)_ = 0.014, *p* > 0.05, η^2^ = 0.000. The results showed that the ACC changes of intentional reasoning were significantly different between the ToM and no-ToM training groups. ToM training had a positive effect on the ACC of intentional reasoning in the ToM training group, and this improvement was maintained until the second post-test, which demonstrates a long-term effect.

The reaction time (RT) interaction was significant, *F*_(2,77)_ = 3.89, *p* < 0.05, η^2^ = 0.048. Simple effect analysis showed that under pre-test conditions, there was no significant difference between the ToM and no-ToM training groups, *F*_(1,78)_ = 2.59, *p* > 0.05, η^2^ = 0.032; at the first post-test, the difference between the ToM and no-ToM training groups was significant, *F*_(1,78)_ = 6.17, *p* < 0.05, η^2^ = 0.015; at the second post-test, the difference between the ToM and no-ToM training groups was also significant, *F*_(1,78)_ = 10.04, *p* < 0.05, η^2^ = 0.058. Moreover, there were significant differences in the three measurement results of the ToM training group, *F*_(2, 77)_ = 4.17, *p* < 0.005, η^2^ = 0.098, but no significant differences in the three measurements for the no-ToM training group, *F*_(2, 77)_ = 1.95, *p* > 0.05, η^2^ = 0.048. Results showed that the RT in the ToM training group became shorter after training, while the RT in the no-ToM training group did not change significantly. ToM training exerted a positive effect on the RT of intentional reasoning in the ToM training group, shortening the RT of intentional reasoning, and this effect was maintained until the second post-test, demonstrating a long-term effect.

In the physical reasoning test, the ACC interaction was no significant, *F*_(2,77)_ = 0.657, *p* > 0.05, η^2^ = 0.017. Simple effect analysis showed that there was no significant difference between the ToM and no-ToM training groups at the pre-test timepoint, *F*_(1,78)_ = 0.027, *p* > 0.05, η^2^ = 0.000; at the first post-test, there was no significant difference between the ToM and no-ToM training groups, *F*_(1,78)_ = 0.958, *p* > 0.05, η^2^ = 0.012; at the second post-test timepoint, there was no significant difference between the ToM and no-ToM training groups, *F*_(1,78)_ = 0.074, *p* > 0.05, η^2^ = 0.001. Moreover, there were no significant differences in the three measurement results of the ToM training group, *F*_(2, 77)_ = 0.34, *p* > 0.005, η^2^ = 0.009, and no significant differences in the three measurements for the no-ToM training group, *F*_(2, 77)_ = 0.45, *p* > 0.05, η^2^ = 0.012. Results showed no significant difference between the two groups measured before and after in the ACC measurement. Physical training had no effect on ACC performance in physical reasoning in the no-ToM training group. This result will be examined in further detail in the discussion section.

RT interaction was not significant, *F*_(2,77)_ = 0.834, *p* > 0.05, η^2^ = 0.011. But paired *t*-test analysis showed that at the pre-test timepoint, there was no significant difference between the ToM and no-ToM training groups, *T*_(39)_ = − 1.337, *p* > 0.05; at the first post-test, the difference between the ToM and no-ToM training groups was significant, *T*_(39)_ = −3.347, *p* < 0.05; at the second post-test, the difference between the ToM and no-ToM training groups was also significant, *T*_(39)_ = − 2.423, *p* < 0.05. Moreover, there were no significant differences in the three measurement results of the ToM training group, *F*_(2, 77)_ = 11.68, *p* < 0.005, η^2^ = 0.233, and no significant differences in the three measurements for the no-ToM training group, *F*_(2, 77)_ = 6.91, *p* < 0.05, η^2^ = 0.152. In physical reasoning, there were significant differences between the two groups in RT performance at all three testing timepoints. Whether this result indicates that physical training has a positive effect on the no-ToM group requires further discussion. Because in the results of the three tests of the ToM training group, the RT also showed a trend of shorter and shorter.

To further examine the impact of training on participant performance in the affective ToM Tasks, a 2 (group: ToM training group, no-ToM training group) × 3 (timepoint: pre-test, post-test 1, post-test 2) repeated measures analysis of variance was conducted.

Results showed that the ACC interaction was not significant in emotional reasoning, *F*_(2,77)_ = 2.52, *p* > 0.05, η^2^ = 0.062. But there were significant differences in the three measurement results of the ToM training group, *F*_(2, 77)_ = 6.404, *p* < 0.005, η^2^ = 0.143, and no significant differences in the three measurements for the no-ToM training group, *F*_(2, 77)_ = 0.165, *p* > 0.05, η^2^ = 0.004. But there were no significant differences in the three measurement results of the ToM training group, *F*_(2, 77)_ = 6.404, *p* < 0.005, η^2^ = 0.143, and no significant differences in the three measurements for the no-ToM training group, *F*_(2, 77)_ = 0165, *p* > 0.05, η^2^ = 0.004. On the affective ToM task, there was no significant difference between the two groups on the pair-wise comparison of the three tests of the ACC for emotional reasoning. But the ToM training group showed significant differences across the three measurements. The results showed that ToM training significantly improved the ACC of ToM training group. And this result shows that physical reasoning training had no effect on ACC.

The RT interaction was not significant, *F*_(2,77)_ = 0.467, *p* > 0.05, η^2^ = 0.006. And there were no significant differences in the three measurement results of the ToM training group, *F*_(2, 77)_ = 0.448, *p* > 0.005, η^2^ = 0.012, and no significant differences in the three measurements for the no-ToM training group, *F*_(2, 77)_ = 2.009, *p* > 0.05, η^2^ = 0.05. Moreover, there were no significant differences in the three measurement results of the ToM training group, *F*_(2, 77)_ = 0.448, *p* > 0.05, η^2^ = 0.012, and no significant differences in the three measurements for the no-ToM training group, *F*_(2, 77)_ = 2.009, *p* > 0.05, η^2^ = 0.05. The results showed that RT performance in emotional reasoning did not differ significantly between the two groups on any of the three measures. The results showed that ToM training had no effect on RT of emotional reasoning in the ToM training group. The reason why it could not help the ToM training group shorten RT is worth being discussed.

In gender reasoning, ACC interaction was not significant, *F*_(2, 77)_ = 0.418, *p* > 0.05, η^2^ = 0.009. And there were no significant differences in the three measurement results of the ToM training group, *F*_(2, 77)_ = 0.903, *p* > 0.05, η^2^ = 0.037, and no significant differences in the three measurements for the no-ToM training group, *F*_(2, 77)_ = 1.786, *p* > 0.05, η^2^ = 0.071. Moreover, there were no significant differences in the three measurement results of the ToM training group, *F*_(2, 77)_ = 0.903, *p* > 0.05, η^2^ = 0.037, and no significant differences in the three measurements for the no-ToM training group, *F*_(2, 77)_ = 1.786, *p* > 0.05, η^2^ = 0.071. In gender reasoning, there was no significant difference in ACC between the two groups across all three tests. The ToM training group also did not show significant differences across the three tests as a whole. The results showed that physical training had no effect on the ACC of gender reasoning in the no-ToM training group. No change in the ACC of gender reasoning in the no-ToM training group is worth being discussed.

RT interaction was not significant, *F*_(2, 77)_ = 0.114, *p* > 0.05, η^2^ = 0.002. And there were no significant differences in the three measurement results of the ToM training group, *F*_(2, 77)_ = 1.103, *p* > 0.05, η^2^ = 0.045, and no significant differences in the three measurements for the no-ToM training group, *F*_(2, 77)_ = 1.51, *p* > 0.05, η^2^ = 0.06. Moreover, there were no significant differences in the three measurement results of the ToM training group, *F*_(2, 77)_ = 1.103, *p* > 0.05, η^2^ = 0.045, and no significant differences in the three measurements for the no-ToM training group, *F*_(2, 77)_ = 1.51, *p* > 0.05, η^2^ = 0.06. In gender reasoning, there was no significant difference in RT between the two groups in any of the three comparison tests. The two groups as a whole also did not show significant differences across the three tests. The results showed that physical training had no effect on the RT of gender reasoning in the no-ToM training group. This result will also be discussed in the discussion section.

## Discussion

In the current study, a new test method was adopted to assess ToM training efficacy. In previous studies of ToM training, questionnaires were used to determine results (Cavallini et al., 2015; Tucci et al., 2016; Caputi et al., 2021). In these studies, significant duplication between the content of the questionnaire and the content of the training existed; hence, familiarity with the content of the test may have resulted in the illusion of improvement. To address this shortcoming, a detection method that was consistent with the training content, but did not exactly duplicate the content, was needed. Therefore, the detection method in this study included intentional reasoning, emotional reasoning, physical reasoning, and gender reasoning, with no duplication between the test content and the training content. It can be seen from the training results that after ToM training, the scores of the ToM group in ACC of intention reasoning and emotional reasoning have been significantly improved, and the performance is better than that of the non-ToM group, and the task scores have been significantly improved in the post-test, which proves that ToM training is effective in improving ToM.

### The key role of mental state conversation

Differences in the language learning experience of deaf people result in a special developmental environment of ToM. Deaf people with hearing parents often grow up in an environment of oral communication, which is limited by their limited oral ability (Lederberg et al., 2013). The limited ability to speak results in individuals within the deaf community having limited opportunities to participate in a variety of conversations, including discussions of different perspectives and the use of terms that describe mental states and a range of other syntactic structures (de Villiers and de Villiers, 2014). Studies have shown that deaf children whose exposure to sign language is delayed have fewer opportunities to talk at home than children who are born deaf and have immediate access to sign language (Peterson and Slaughter, 2006). Success on the false belief task shows a significant delay (Peterson and Siegal, 1999). The reason why an environment that contains conversations about mental states is so important to the development of ToM may be that it draws attention to the inner states of others and shapes their expectations or experiences of interpersonal events (Slaughter and Peterson, 2012). Exposure to environments that contain conversations about mental states not only encourages individuals to pay closer attention to mental states, but can also help individuals learn how to better spot specific mental states, and thus have a better understanding of what is happening in a particular social context (Apperly, 2011). Many studies have shown that mental state conversation is the key factor determining individual differences in ToM (de Rosnay and Hughes, 2006). Mental state conversation can predict ToM development (Lecce et al., 2014). As a result, a large amount of mental state conversation was incorporated into this training, including reading material and interactive content. The deaf college students in the current study were effectively immersed in the environment of mental state conversation in an effort to improve their ToM level. Deaf college students have improved their theory of mind, which is consistent with previous research results (Bianco et al., 2021; Lombardi et al., 2022).

### The role of explanation in training

In verbal conversation, explanation is crucial (Melot and Angeard, 2003). Giving explanations in a conversation can help to show the relationship between a character’s inner state and outer behavior (Dunn et al., 1991). A study by Marschark, Spencer, Adams, and Sapere showed that hearing adults are more likely to control conversation when a child is hearing impaired, and to avoid confusion, deaf children rarely mention their own doubts, misunderstandings, or incorrect ideas (Marschark et al., 2011). Even children who receive cochlear implants in early childhood are less likely to be exposed to the informal causal explanatory conversations about people associated with early false belief success that occur in families without hearing deficits (Peterson and Slaughter, 2003). Studies that ask deaf children to explain rules of school behavior have found that deaf children perform significantly worse than non-deaf children (Rachford and Furth, 1986). Calderon and Greenber similarly found that deaf children often misunderstand or ignore the “why” question in conversation. Researchers believe that limited interpretation and limited experience deprive many deaf children of legitimate opportunities to learn how to understand others (Calderon and Greenberg, 2003). Taking the above into account, the primary reason for the success of the intervention in the current study is primarily due to the interactions participants had with trainers. The trainer consciously reminded participants to explain their choices, and to explain the mental state and purpose of the behavior of the hero in the story. When the participant demonstrated difficulty in explanation, the trainer invited other participants to assist in the explanation and help to craft a correct explanation. Unfortunately, interpretation was not examined as a separate factor, which will be considered in subsequent studies.

### Training effects of training on ToM

Intentional and emotional reasoning was primarily improved in the current study through the analysis, sharing, and explanation of ToM stories. In contrast, the no-ToM training group only practiced physical reasoning. In cognitive ToM, intentional reasoning ability of the ToM training group was improved after training. ACC was increased and RT was decreased.

Interestingly, although the no-ToM training group was trained in physical reasoning, there was no significant difference in ACC changes in physical reasoning before and after training in the no-ToM training group, with only RT showing a significant decline. There are two possible reasons why ACC did not change in the no-ToM training group before and after training. One reason may be related to the content of the training, which focused on the change of the physical characteristics of the stimuli, such as shape and size, and did not include the outcome of the stimuli following manipulation, such as a hat being blown away by the wind or a ball being bounced away. One reason may be that the difficulty of the physical reasoning included in the test was simple enough for deaf college students to perform well without training. The shortening of RT in both training groups may be due to the fact that participants in both groups were more confident in answering correctly when faced with physical reasoning, resulting in a practice effect, which was demonstrated by steadily increasing speed after several tests.

ToM training has a positive effect on ACC of emotional reasoning, but no effect on RT. ToM training included emotional reasoning training. Participants expressed their emotions and simulated their expressions by imitating the scene portrayed in the story, which helped participants identify representations of various emotional states, thus improving ACC of emotional reasoning. However, this training demonstrated no effect on reducing emotional reasoning RT, possibly because emotional judgment is a form of complex reasoning; complex expression can produce different explanations for causes. Some of the expression pictures in the test material may have been difficult to distinguish in a short time, even after multiple measurements. Therefore, all three tests required participants to spend a certain amount of time to identify the details of the emoticons before they could make judgments, which resulted in no change in RT in the ToM training group among the three tests. Compared with the other three tasks, gender judgment should be the easiest task, and both groups consistently scored higher on gender reasoning in the three tests. This could explain why the two groups did not produce significant differences in both ACC and RT of gender reasoning.

It is found that ToM training has a long term effect on ACC and RT of intentional reasoning, a long term effect on ACC of emotional reasoning, and no long term effect on RT of emotional reasoning. These results suggest that ToM training has a long-term effect on both cognitive ToM and affective ToM. The reason that training has no long-term effect on RT of emotional reasoning is understandable, because ToM training has no effect on emotional reasoning. The reasons for this lack of impact have been discussed earlier.

### Limitations and prospects

There are several limitations to this study. The first relates to the participants themselves; participants in this study were all deaf college students from a single school. Future studies can expand the scope of participants, as the level of ToM in the deaf community requires attention. The second factor is related to improving the level of ToM. In this study, interpretation was integrated into the training process as a key point, but when analyzing the results, interpretation was not extracted as a factor for separate analysis to see the magnitude of effect it exerted in the training process. Future research can extract several key factors to improve the level of ToM and construct models to compare the effects of factors. The final limitation relates to the timing of the post-test; since the training was scheduled according to the learning progress of the school, the second post-test can only be scheduled 2 months later, and there was no time to schedule a third post-test to further examine the long-term effects of training. In the future, a third post-test can be administered the next semester. Unfortunately, due to the problem of the number of participants, the hearing status was not analyzed as a single variable in this study, and the relationship between the hearing status and training effect was investigated. We will try to add it into the study in the future.

## Summary

To summarize, the addition of ToM training involving mental state conversation can improve ToM level. This research training demonstrated a certain impact on the ability of ToM, including enhancement of intentional and emotional reasoning, and can be implemented in the deaf community to help deaf individuals improve their level of ToM and better adapt to society.

## Conclusion

This study designed a training course to improve the level of deaf college students’ ToM. The results show that through ToM training, the level of ToM (including cognitive ToM and affective ToM) of deaf college students was significantly improved. Furthermore, the effects of the training were still present 2 months later. This shows that mental state conversation is an important factor restricting ToM level in deaf individuals.

## Data availability statement

The raw data supporting the conclusions of this article will be made available by the authors, without undue reservation.

## Ethics statement

The studies involving humans were approved by Ethics Committee of Tianjin Normal University. The studies were conducted in accordance with the local legislation and institutional requirements. The participants provided their written informed consent to participate in this study.

## Author contributions

YW: Formal analysis, Investigation, Methodology, Writing – original draft, Writing – review & editing. XL: Conceptualization, Writing – review & editing. SZ: Data curation, Software, Validation, Writing – review & editing.
